# Integrating network pharmacology and transcriptomics to reveal the therapeutic effect of Long Mu Ning Xin Decoction on attention-deficit/hyperactivity disorder by regulating cAMP and PI3K/AKT pathways

**DOI:** 10.3389/fphar.2026.1744709

**Published:** 2026-01-30

**Authors:** Xiaodan Ren, Lele Ding, Yonghong Jiang

**Affiliations:** 1 Pediatrics, Longhua Hospital Affiliated to Shanghai University of Traditional Chinese Medicine, Shanghai, China; 2 Longhua Clinical Medical College, Shanghai University of Traditional Chinese Medicine, Shanghai, China

**Keywords:** attention-deficit/hyperactivity disorder, cAMP signaling pathway, Long Mu Ning Xin decoction, network pharmacology, PI3K-Akt signaling pathway, spontaneously hypertensive rats, transcriptomics

## Abstract

**Background:**

Attention-deficit/hyperactivity disorder (ADHD) is a prevalent neurodevelopmental disorder in children. Long Mu Ning Xin Decoction (LMNXD) shows established clinical efficacy against ADHD, yet its mechanistic basis is not fully elucidated.

**Objective:**

This study investigates the therapeutic potential of LMNXD for ADHD and explores its underlying mechanisms of action.

**Methods:**

Thirty spontaneously hypertensive rats (SHRs/NCrl) were randomly divided into five groups: a model (SHR) group, low-, medium-, and high-dose LMNXD (LMNXD-LD, LMNXD-MD, LMNXD-HD)groups, and a methylphenidate hydrochloride (MPH) group. Additionally, six Wistar Kyoto (WKY/NCrl) rats were designated as the control group.Behavioral performance was assessed using the open field test and Morris water maze. The expression levels of glial fibrillary acidic protein (GFAP), dopamine deceptor D1 (DRD1), and brain-derived neurotrophic factor (BDNF) in the rat hippocampus, prefrontal cortex (PFC), and striatum were evaluated by immunofluorescence, immunohistochemistry, and Western blot. Potential targets and mechanisms were explored through transcriptomic sequencing and network pharmacology, with subsequent validation by reverse transcription quantitative polymerase chain reaction (RT-qPCR).

**Results:**

Compared to the SHR group, LMNXD ameliorated hyperactivity, impulsivity, deficits in spatial memory and learning ability in SHR/NCrl rats. It also effectively reduced GFAP expression in the hippocampus while increasing DRD1 expression in the PFC and BDNF levels in the striatum. Network pharmacology predicted that LMNXD might alleviate ADHD by acting on pathways including phosphatidylinositide 3-kinase-Akt (PI3K-Akt), calcium signaling, and cyclic adenosine monophosphate (cAMP) signaling. Consistent with this prediction, transcriptomic analysis of rat hippocampi showed that LMNXD influences the cAMP and PI3K-Akt signaling pathways, as well as serotonergic and cholinergic synapses. RT-qPCR further confirmed that LMNXD likely exerts its therapeutic effect by regulating the mRNA expression of ATPase Plasma Membrane Ca^2+^ Transporting 4 (ATP2B4), Glutamate Ionotropic Receptor NMDA Type Subunit 3A (GRIN3A), Oxytocin Receptor (OXTR), Collagen Type VI Alpha 2Chain (COL6A2), and Integrin Subunit Alpha 1 (ITGA1) within the cAMP andPI3K-Akt pathways.

**Conclusion:**

LMNXD may ameliorates hyperactive-impulsive behaviors and improves spatial memory and learning in SHRs/NCrl rats by modulating ATP2B4, GRIN3A, OXTR, COL6A2, and ITGA1 within the cAMP and PI3K-Akt signaling pathways. This intervention also upregulates DRD1 and BDNF expression while downregulating GFAP levels.

## 1 Introduction

Attention-deficit/hyperactivity disorder (ADHD) is a neurodevelopmental condition defined by persistent inattention, hyperactivity, and impulsivity (Rajaprakash and Leppert, 2022). It affects approximately 8.0% of children and adolescents worldwide, with a prevalence in boys that is twice that observed in girls (Ayano et al., 2023). Moreover, an estimated 70%–80% of individuals with ADHD experience comorbid psychiatric and non-psychiatric disorders throughout their lives, such as oppositional defiant disorder, conduct disorder, bipolar disorder, anxiety disorders, substance use disorders, autism spectrum disorder, and dyslexia (Ribasés et al., 2023). Research further indicates that ADHD elevates the risk of criminal behavior in adolescence and adulthood. Without timely intervention, symptoms persist into adulthood for roughly one-quarter of affected children, often resulting in impaired social adaptation, interpersonal difficulties, increased suicidality, and elevated antisocial behavior, which collectively impose a substantial burden on individuals, families, and society (Riglin et al., 2023).

The core pathology of ADHD involves dysregulated dopamine (DA) and norepinephrine (NE) signaling in the prefrontal-striatal circuit, which diminishes synaptic plasticity and compromises information transmission efficiency (Quinteroet al., 2022; Anderson et al., 2024). This manifests as significant deficits in attention, executive function, and motivation control. Additional studies have identified neuroinflammation as a key driver in the pathogenesis of ADHD. Neuroinflammatory processes can activate glial cells, inhibit the synthesis and release of DA and NE, and impair synaptic plasticity in the hippocampus–prefrontal cortex pathway, thereby exacerbating the pathological progression of the disorder (Martins et al., 2023). First-line pharmacological treatments for ADHD, such as methylphenidate hydrochloride (MPH) and amphetamine, function by inhibiting the reuptake of DA and NE, thereby increasing their concentrations in the synaptic cleft and improving attention and executive functions (Pitzianti et al., 2020). Their clinical use, however, is complicated by two principal issues: frequent adverse effects including insomnia, appetite suppression, and depression (Storebø et al., 2015); and a risk of abuse owing to their stimulant properties, with long-term or improper use potentially leading to addiction and psychological dependence (Chamakalayil et al., 2020). Therefore, the identification of safe and effective alternative therapeutics has become an urgent priority.

As a complementary and alternative therapy for ADHD, traditional Chinese medicine (TCM) has demonstrated favorable efficacy and safety. The National Administration of Traditional Chinese Medicine of China has recognized ADHD as a condition for which TCM provides unique therapeutic advantages (Liu, et al., 2024). According to TCM theory, the etiology of ADHD is attributed to visceral dysfunction and an imbalance between yin and yang. We further summarize its pathogenesis as a deficiency of kidney yin coupled with hyperactivity of heart yang. Long Mu Ning Xin Decoction (LMNXD) is a modified TCM formula derived from the classical prescription Tian Wang Bu Xin Dan, which holds a national invention patent (No. ZL202410812364.3). Having been used clinically for nearly a decade, this formulation has demonstrated reliable therapeutic efficacy. Earlier research established that Tian Wang Bu Xin Dan alleviates clinical symptoms and slows disease progression in ADHD patients (Yao et al., 2020), thereby providing atheoretical and practical basis for LMNXD. The formula contains fifteen medicinal components: raw Os Draconis, raw Concha Ostreae, Magnetitum, Rehmanniae Radix Praeparata, Plastrum Testudinis, Rhizoma Acori Tatarinowii, Radix Polygalae Praeparata, Fructus Alpiniae Oxyphyllae, Radix Ophiopogonis, Radix Asparagi, Radix Angelicae Sinensis, Poria cum Radix Pini, Fructus Schisandrae, *Triticum aestivum*, Rhizoma Dioscoreae. It acts as a compound preparation to nourish yin, suppress yang, harmonize the heart and kidney, and calm the mind. Nevertheless, the precise mechanisms and targets through which LMNXD produces these effects are not yet fully understood.

With the advancement of systems biology, network pharmacology and transcriptomics have provided new paradigms for elucidating the mechanisms of TCM in treating ADHD. Network pharmacology supports the systematic identification of key targets and pathways implicated in ADHD (Song et al., 2022), whereas transcriptomics provides a comprehensive analysis of gene expression changes within key brain regions of ADHD models after TCM intervention (You et al., 2025). Integrating these two approaches enables multi-level validation, spanning from macroscopic networks to microscopic genes, and establishes a systematic, reproducible framework for elucidating the molecular mechanisms of TCM in ADHD treatment (Li et al., 2022).

Spontaneously hypertensive rats (SHRs/NCrl), sourced from Charles River in Germany, demonstrate high congruence with human ADHD across multiple dimensions, including behavioral phenotypes, responses to first-line ADHD medications, and underlying neurotransmitter, circuitry, and gene expression profiles (Bogdańska-Chomczyk et al., 2024). This strain is therefore considered a robust experimental model for studying ADHD pathophysiology, screening potential therapeutics, and evaluating interventions. The Wistar Kyoto substrain obtained from Harlan in the UK (WKY/NHsd) provides the most appropriate control (Sagvolden et al., 2009).

**FIGURE 1:**
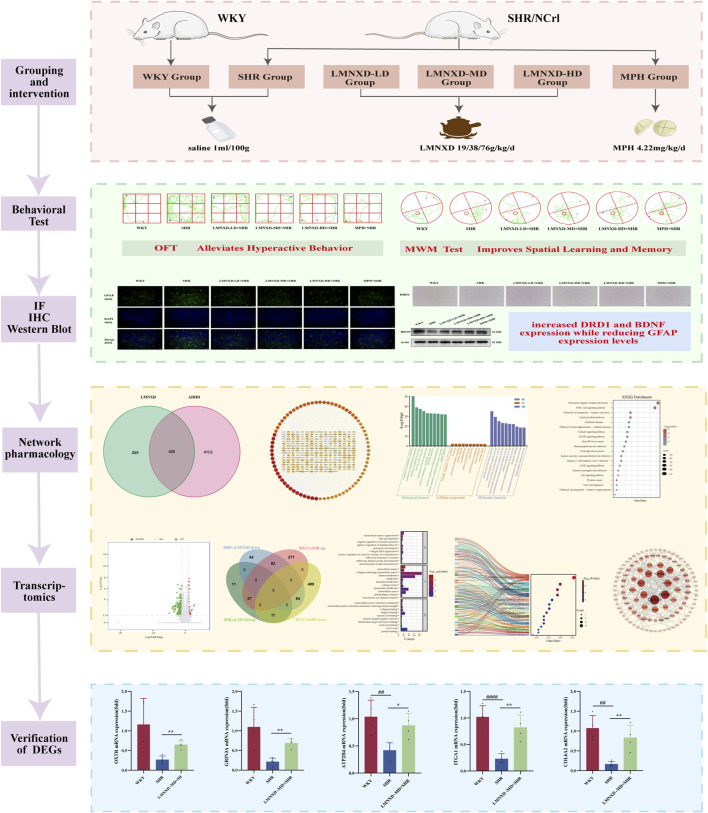
Graphical abstract.

Therefore, this study employed SHRs/NCrl rats as an animal model of ADHD and WKY rats as blank controls. We evaluated the therapeutic effects of LMNXD on SHRs/NCrl rats and investigated its mechanism of action in ADHD using network pharmacology and transcriptomics (Figure 1).

## 2 Materials and methods

### 2.1 Chemicals and reagents

Methylphenidate hydrochloride extended-release tablets (Batch No.: HJ20225002) were purchased from Xi’an Janssen Pharmaceutical Ltd (Xi’an, China). Primary antibodies against glial fibrillary acidic protein (GFAP, 60190-1-Ig), dopamine deceptor D1 (DRD1, 17934-1-Ap), and BDNF (66292-1-Ig) were obtained from Proteintech (Wuhan, China). RNA was extracted using the EZB-RN10 kit from EZBioscience (Shanghai, China). Reverse transcription and quantitative PCR were performed respectively using the reverse transcription (kitRR092A) and the QuantityNova SYBR Green PCR kit (RR420A), both of which were purchased from Takara Bio (Beijing, China).

### 2.2 Preparation and composition identification of LMNXD

LMNXD comprises 15 herbal components: raw Os Draconis (15 g), raw Concha Ostreae (15 g), Magnetitum (15 g), Rehmanniae Radix Praeparata (9 g), Plastrum Testudinis (9 g), Rhizoma Acori Tatarinowii (9 g), Radix Polygalae Praeparata (9 g), Fructus Alpiniae Oxyphyllae (9 g), Radix Ophiopogonis (9 g), Radix Asparagi (9 g), Radix Angelicae Sinensis (9 g), Poria cum Radix Pini (9 g), Fructus Schisandrae (6 g), *Triticum aestivum* (15 g), Rhizoma Dioscoreae (15 g). All herbs were purchased from Longhua Hospital, Shanghai University of Traditional Chinese Medicine, and processed into granular form.

The chemical constituents of LMNXD were analyzed by ultra-high performance liquid chromatography-Orbitrap mass spectrometry (UHPLC-Orbitrap-MS). To prepare the LMNXD extract, 300 μL of supernatant was combined with 1,000 μL of an extraction solution (methanol:acetonitrile:water = 2:2:1, v/v/v) containing isotope-labeled internal standards. This mixture was vortexed for 30 s, sonicated in an ice bath for 5 min, incubated at −20 °C for 1 h, and centrifuged at 12,000 rpm for 15 min at 4 °C. The resulting supernatant was passed through a 0.22 μm membrane filter to yield the final filtrate. Chromatographic separation was carried out on a Vanquish UHPLC system (Thermo Fisher Scientific) fitted with a Phenomenex Kinetex C18 column (2.1 mm × 100 mm, 2.6 μm). The mobile phase comprised 0.01% acetic acid in water (solvent A) and a 1:1 (v/v) mixture of isopropanol and acetonitrile (solvent B). Mass spectrometric detection employed an Orbitrap Exploris 120 MS operating in both positive and negative ion modes, with the following key parameters: sheath gas and auxiliary gas flow rates of 50 and 15 Arb, respectively; a capillary temperature of 320 °C; full MS resolution of 60,000; MS/MS resolution of 15,000; and spray voltages of ±3.8/3.4 kV. Data were processed using ProteoWizard and an in-house R software package integrated with the Biotree TCM/BT-HERB database.

### 2.3 Experimental animals and procedures

Thirty 4-week-old male SHR/NCrl rats, representing an age equivalent to 9-year-old human males (https://www.taletn.com/rats/age/), and six age-matched male WKY/NCrl rats, were sourced from Beijing Vital River Laboratory Animal Technology Co., Ltd. (SCXK (Jing) 2021-0006). All animals were housed under standard conditions at 23 °C ± 2 °C, with relative humidity maintained between 50% and 60% and a 12-h light/dark cycle. Food and water were available *ad libitum*. All experimental procedures strictly followed the guidelines of the Chinese Institute of Laboratory Animal Sciences and internationally recognized principles for the care and use of laboratory animals. The experimental protocol was approved by the Institutional Animal Care and Use Committee of SHRM (Approval No.: LL-2025-299).

SHR/NCrl rats were randomly divided into five groups: the model group (SHR), the LMNXD low-dose group (LMNXD-LD), the LMNXD medium-dose group (LMNXD-MD), the LMNXD high-dose group (LMNXD-HD), and the MPH group (4.22 mg/kg/d). WKY/NCrl rats served as the blank control group.The clinically effective pediatric dose of LMNXD is 162 g/d. The dose administered to the animals was based on the body surface area of a 9-year-old (body mass 26 kg) child (0.991 m^2^, Stevenson’s formula = 0.0061×height (cm)+0.0128×body weight (kg)-0.1529) and 4-week-old (body mass 60 g) SHR pups body surface area (0.013947 m^2^, Meeh-Rubner formula = 9.1 × body weight (g)^2⁄3^/10,000) were converted in parallel (Li et al., 2024). This conversion yielded a clinically equivalent dose of 38 g/kg/d for SHR/NCrl rats. The LMNXD-MD group received this clinically equivalent dose (38 g/kg/d), the low dose was set to half the clinically effective dose (19 g/kg/d), and the high dose was twice the clinically effective dose (76 g/kg/d). The TCM granules were dissolved in distilled water before administration. Intragastric administration was conducted at 1 mL/100 g body weight, twice daily (8:00–9:00 a.m. and 14:00–15:00 p.m.), over four consecutive weeks. The SHR and WKY groups received an equivalent volume of normal saline *via* intragastric administration.

### 2.4 Behavioral tests

#### 2.4.1 Open field test (OFT)

The Open Field Test (OFT) was performed before and after the treatment (Ding et al., 2022). The setup included a black square arena (100 cm × 100 cm × 50 cm), a camera, a peripheral frame matching the arena’s dimensions, a computer, and specialized animal behavior analysis software. Before testing began, all animals were acclimatized to the behavioral laboratory for 1 hour. Each rat was placed into the arena from a fixed starting position. A video camera recorded and tracked their movement trajectories, and the locomotor paths were subsequently analyzed using SuperMaze animal behavior analysis software (Version 3.3.0.0, Shanghai Xinruan Information Technology Co., Ltd). The primary outcome measures were total distance traveled, average velocity, and immobility time (n = 6 rats per group).

#### 2.4.2 Morris water maze (MWM) test

The Morris water maze (MWM) test commenced on the 22nd day of treatment and continued for six consecutive days (Sun et al., 2024). The MWM experimental setup consists of a circular black water tank with a diameter of 150 cm and a depth of 50 cm, divided into four quadrants and filled with water maintained at a temperature of 24 °C ± 1 °C. A circular platform, measuring 12 cm in diameter, is submerged approximately 1 cm below the water surface at the center of the southwest quadrant. Additionally, 80 mL of black ink was introduced to create a contrast against the rats’ natural colouration.

The concealed platform test was administered from the first to the fifth day, with one trial conducted in each of the four quadrants daily, allowing for a 15-min interval between tests. The primary metric is the escape latency, defined as the time taken for rats to locate the hidden platform within a 60-s timeframe. Rats were allotted 60 s to locate the hidden platform and were permitted to rest on it for 10 s. If a rat failed to find the platform within the designated time, it was guided to the platform and permitted to rest for 10 s, with the escape latency recorded as 60 s.

On the sixth day, a spatial exploration experiment was conducted without the platform, allowing each rat to swim freely for 60 s. The number of times rats crossed the platform location within the 60 s, the duration spent in the target quadrant (where the platform was previously located), and the total swimming distance were all documented. The maze was enclosed with black blackout cloth to minimize external environmental interference (N = 6 rats in each group).

### 2.5 Sample collection and processing

After behavioral testing, the rats were fasted for 12 h and anesthetized with Zoletil^®^ 50 (50 mg/kg). The animals were then decapitated using a guillotine. The PFC, striatum, and hippocampus were rapidly dissected on ice with glass needles after removing the skin and skull. Any residual blood or hair was carefully rinsed away with cold phosphate-buffered saline (PBS). The dissected tissues were immediately frozen in liquid nitrogen and stored at −80 °C.

### 2.6 Immunohistochemistry and immunofluorescence

For immunohistochemistry, three rats were randomly selected from each group. PFC tissue was fixed in 4% paraformaldehyde, embedded in paraffin, and sectioned at a thickness of 3–4 μm. The sections underwent deparaffinization, antigen retrieval, endogenous peroxidase quenching, and blocking with 3% BSA. They were then incubated overnight at 4 °C with a primary antibody DRD1 (1:100). Following PBST washes, the sections were incubated for 2 hours at room temperature with an HRP-labeled goat anti-rabbit IgG secondary antibody and developed with DAB, which produced a brown-yellow color indicative of positive staining. Nuclei were counterstained with hematoxylin for 3 minutes before the sections were dehydrated and mounted. Finally, the sections were scanned under a microscope, and images from three randomly chosen fields were captured for DRD1 expression analysis using ImageJ software (Mizote et al., 2024).

Hippocampal tissues from three randomly selected rats per group were processed for immunofluorescence staining. Sections were dewaxed, underwent antigen retrieval, and blocked with FBS. They were then incubated overnight at 4 °C with GFAP antibody (1: 100). After washing, the sections were treated with a fluorophore-conjugated secondary antibody and counterstained with DAPI to label nuclei. Fluorescence microscopy was used to scan the sections, and three random high-power fields per sample were selected for evaluation. The mean value from these three fields served as the final result for each sample. GFAP expression was quantified using ImageJ software.

### 2.7 Western blot analysis

Striatum tissues from six randomly selected rats per group were analyzed by Western blot. The tissues were homogenized and lysed before centrifugation to collect the supernatant, whose protein concentration was measured with a BCA protein quantification kit (P0012, Beyotime, China). Proteins were separated using 10% SDS-PAGE, loading 40 μg per lane. After electrophoresis and transfer, the membranes were blocked for 30 min with a rapid protein-free blocking solution (BL1032B). They were then incubated overnight at 4 °C with the appropriate primary antibodies. Following washes, the membranes were incubated with corresponding secondary antibodies for 1 hour at room temperature. Protein bands were visualized *via* ECL detection, and the grayscale values of target bands were quantified using ImageJ software.

### 2.8 Network pharmacology

The structures and SMILES identifiers for the 70 compounds identified in the LMNXD mass spectrometry analysis were retrieved from the PubChem database and ChemDraw. These compounds then underwent ADME screening *via* the SwissADME platform. Their structures were submitted to the SwissTargetPrediction and STITCH databases to predict potential targets, which were subsequently compiled and deduplicated to yield the final target set. Disease targets related to ADHD were collected by querying the OMIM, GeneCards, DrugBank, and PharmGKB databases with the keyword “Attention-deficit/hyperactivity disorder.” The intersection of LMNXD and ADHD targets was defined as the potential therapeutic target genes of LMNXD for ADHD. A protein-protein interaction (PPI) network for these overlapping targets was built using the STRING database. The resulting network file was imported into Cytoscape for visualization. Finally, a “component-target-disease-pathway” network was constructed in Cytoscape.

### 2.9 Transcriptomics

Hippocampal tissues were randomly selected from four rats in the WKY group, SHR group, and LMNXD-M group for transcriptomic analysis. Total RNA was extracted using TRIzol reagent. The integrity and quantity of RNA were assessed using the Agilent 2100 bioanalyzer (Agilent Technologies, Santa Clara, CA, USA). Purity and concentration were simultaneously determined using the NanoDrop ND-1000 (NanoDrop, DE, USA). Total RNA was used as the starting material for mRNA enrichment and fragmentation, followed by cDNA synthesis. The cDNA underwent end repair, adapter ligation, size selection, and amplification to construct the libraries, which were then quality-controlled using Qubit, Agilent 2100, and qRT-PCR. After passing quality control, these libraries were sequenced on the Illumina Novaseq 6000 platform, generating 150-bp paired-end reads. Raw data were converted into FASTQ files, and clean reads were obtained after filtering out low-quality sequences. HISAT2 was used to align the reads to the reference genome. Novel transcripts were predicted with StringTie, and featureCounts quantified reads mapped to each gene. Principal Component Analysis (PCA) was conducted on the gene expression values (FPKM) of all samples to evaluate biological replicates. Differential expression analysis was performed using DESeq2 to identify significantly differentially expressed genes (DEGs), with thresholds established at |log2(Fold Change)| ≥ 1 and an adjusted *P*-value (*P*adj) ≤ 0.05.

### 2.10 GO and KEGG analysis

To investigate the mechanisms underlying LMNXD treatment for ADHD, we conducted an integrated and annotated analysis of network pharmacology targets and transcriptomic DEGs using DAVID (https://david.ncifcrf.gov/). This included functional annotation through Gene Ontology (GO) and Kyoto Encyclopedia of Genes and Genomes (KEGG) enrichment analyses. The GO and KEGG enrichment results were then visualized with bioinformatics tools.

### 2.11 Real-time fluorescence quantitative reverse transcription PCR (RT-qPCR)

Total RNA was extracted from 40 mg of hippocampal tissue with Trizol reagent. cDNA was synthesized from the extracted RNA using the Invitrogen First Strand cDNA Synthesis Kit (RR092A). Primers targeting ATPase Plasma Membrane Ca^2+^Transporting 4 (ATP2B4), Glutamate Receptor Ionotropic NMDA 3A (GRIN3A), Oxytocin Receptor (OXTR), Collagen Type VI Alpha 2Chain (COL6A2), and Integrin Subunit Alpha 1 (ITGA1) were designed *via* NCBI Primer-BLAST based on GenBank sequences, with their specific sequences provided in Table 1. Amplification of cDNA was then carried out with a SYBR Green PCR kit under the following cycling protocol: 95 °C for 30 s, then 40 cycles of 95 °C for 5 s and 60 °C for 10 s. The detailed composition of the PCR reaction mixture is specified in Table 2. Relative mRNA expression levels for the target genes were calculated according to the 2^−ΔΔCT^ method. The RT-qPCR analysis included four biological replicates per experimental group.

**TABLE 1:** The list of qPCR primers.

Gene	Forward sequence	Reverse sequence
ATP2B4 (144 bp)	GCC​AGC​ATC​ACT​AGG​TGG​TT	GAT​GGC​TAG​GCC​TCT​CTC​TT
OXTR (86 bp)	TAC​CGT​CAC​CAC​CGA​GAA​ATC	TGC​CTG​TCC​TCT​CCC​TGA​GT
GRIN3A (101 bp)	CTG​CAC​ACG​AGT​CAG​AGG​TT	AGG​TTG​GAC​CTC​TTC​TCC​ACA
COL6A2 (125 bp)	CTT​CCC​TGC​CAA​ACA​GAT​GAA​C	GCG​TTT​AGC​AAC​TGC​TGG​AT
ITGA1 (119 bp)	TCA​GTC​CAC​GAA​CAC​ATT​CCC	TGC​TGG​GAC​TTG​ACG​ATC​AG
GAPDH (265 bp)	TTG​TGA​AGC​TCA​TTT​CCT​GGT​ATG	TGG​TAT​TCG​AGA​GAA​GGG​AGG

**TABLE 2:** Reaction system of RT-qPCR.

Reagents	Volume (μL)
TB Green Premix Ex (Tli RNaseH Plus)2X	10
Forward Primer (10 μM)	0.4
Reverse Primer (10 μM)	0.4
ROX Reference Dye (50X)	0.4
cDNA	2
Sterilized water	6.8

### 2.12 Statistical analyses

Data analysis was performed with SPSS 25.0 software, and measurement data are presented as the mean ± standard deviation. For the data of each index, we first performed the normality test and the homogeneity of variance test. Intergroup comparisons employed one-way analysis of variance (ANOVA), while multiple comparisons used the LSD and Dunnet methods. Datasets involving treatments and time points were analyzed with a two-factor repeated measures analysis of variance. A *P* value <0.05 was considered statistically significant. Graphs were generated using GraphPad Prism 8.0 software.

## 3 Results

### 3.1 Identification of LMNXD components

**FIGURE 2:**
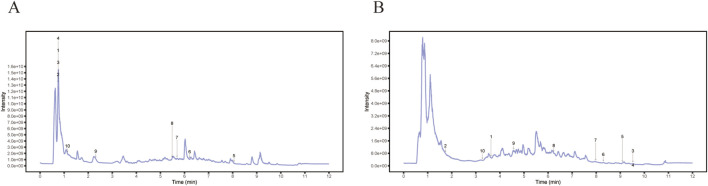
The qualitative and quantitative analyses of LMNXD was subjected to comprehensive profiling using UHPLC-OE-MS. **(A)** Positive ion mode of LMNXD TIC peaks. **(B)** Negative ion mode of LMNXD TIC peaks.

A comprehensive analysis using UHPLC-OE-MS qualitatively and quantitatively determined the chemical constituents of LMNXD, leading to the identification of 70 distinct chemical structures (Supplementary Table 1). These constituents comprised seven flavonoids, such as Liquiritigenin, six phenylpropanoids including N-p-trans-Coumaroyltyramine, and six phenolic acids like 4-Hydroxybenzaldehyde. The analysis also identified five terpenoids, for example, Nootkatone, four lignans such as Schisandrin B, three alkaloids including 3-Pyridinemethanol, and 38 other components (Figures 2A,B).

### 3.2 LMNXD alleviates hyperactive behavior in SHR/NCrl rats

The body weights of the rats in each group are shown in Table 3, and LMNXD had no significant effect on the weight gain of the rats.

**TABLE 3:** Changes in body weight of rats in each group.

Group	0 Week (g)	2 Week (g)	4 Week (g)
WKY	91.33 ± 3.4	142.47 ± 3.77	207.83 ± 7.61
SHR	90.04 ± 2.81	146.15 ± 4.38	214.06 ± 4.96
LMNXD-LD+SHR	86.47 ± 5.03	145.12 ± 3.38	208.52 ± 6.18
LMNXD-MD+SHR	87.3 ± 4.74	141.87 ± 3.9	210.48 ± 3.8
LMNXD-HD+SHR	88.77 ± 3.96	143.74 ± 7.74	207.3 ± 7.3
MPH+SHR	87.6 ± 5.07	144.07 ± 4.2	210.16 ± 7.09

**FIGURE 3:**
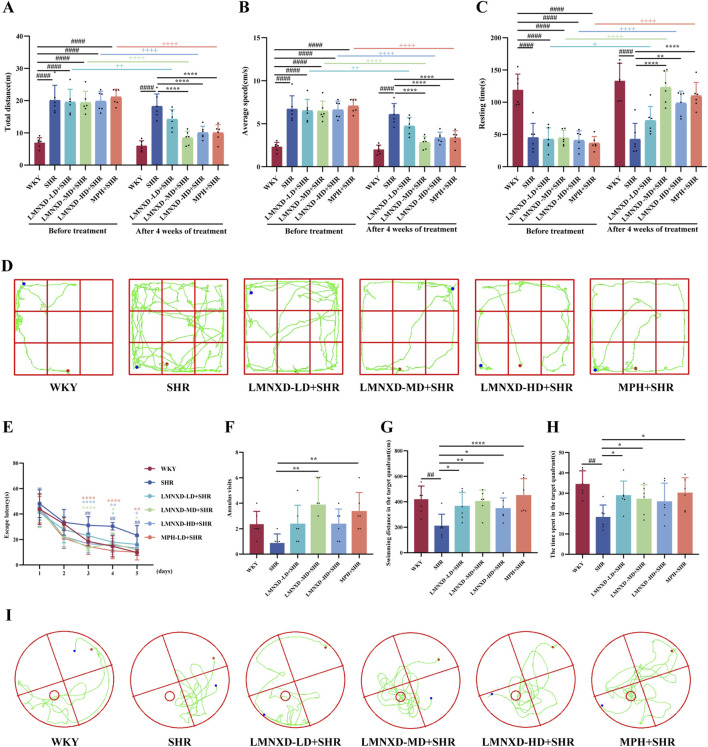
Behavioral performance of rats in the OFT and MWM test. **(A)** Moving total distance in the OFT. **(B)** Average speed in the OFT. **(C)** Resting time in the OFT **(D)** Representative trajectories of each group of rats in the OFT. **(E)** Escape latency in the MWM test. **(F)** Number of annulus visits in MWM test. **(G)** Swimming distance in the target quadrant in the MWM test. **(H)** Time spent in the target quadrant in the MWM test. **(I)** Representative trajectories of each group in the MWM test. All data are presented as mean ± SEM. ^##^ *p* < 0.01 and ^####^ *p* < 0.0001 compared with the WKY group. *p < 0.05, **p < 0.01, and ****p < 0.0001 compared with the SHR group. ^+^ *p* < 0.05, ^++^ *p* < 0.01, and ^++++^ *p* < 0.0001 compared with the same group before treatment.

The OFT assessed the effects of LMNXD on rat locomotor activity by measuring the total distance moved, average speed, and resting time. Before treatment, the SHR, LMNXD-LD + SHR, LMNXD-MD + SHR, LMNXD-HD + SHR, and MPH + SHR groups exhibited significantly greater total distance and average speed (*P* < 0.0001, Figures 3A,B) and significantly less resting time (*P* < 0.0001, Figure 3C) than the WKY group.

Following 4 weeks of treatment, the SHR group continued to display significantly elevated total distance and average speed (*P* < 0.0001, Figures 3A,B) and significantly reduced resting time (*P* < 0.0001, Figure 3C) relative to the WKY group. By contrast, the LMNXD-MD + SHR, LMNXD-HD + SHR, and MPH + SHR groups showed significant reductions in total distance and average speed (*P* < 0.0001, Figures 3A,B) and a significant increase in resting time (Figure 3C) compared to the SHR group. Furthermore, relative to their pre-treatment baselines, the LMNXD-LD + SHR, LMNXD-MD + SHR, LMNXD-HD + SHR, and MPH + SHR groups demonstrated significantly decreased total distance and average speed (Figures 3A,B) and significantly increased resting time (Figure 3C) after the 4-week treatment period. These findings indicate that LMNXD effectively ameliorates hyperactive and impulsive behaviors in SHR/NCrl rats, with definitive efficacy at medium and high doses. Representative movement trajectories for each group after administration are presented in Figure 3D.

### 3.3 LMNXD improves spatial learning and memory in SHR/NCrl rats

The MWM test assessed the effect of LMNXD on spatial learning and memory in SHRs/NCrl rats. From day 3 onward, the escape latency was significantly shorter in the LMNXD-MD + SHR, LMNXD-HD + SHR, MPH + SHR, and WKY groups than in the SHR group (Figure 3E). On day 4, the LMNXD-LD + SHR group also displayed a significantly shorter escape latency than the SHR group (*P* < 0.05, Figure 3E).

During the spatial probe trial on day 6, all four treatment groups showed an increased number of platform crossings relative to the SHR group, but significant differences emerged only for the MPH + SHR and LMNXD-MD + SHR groups (*P* < 0.01, Figure 3F). The SHR group spent significantly less total distance and time in the target quadrant than the WKY group (*P* < 0.01, Figures 3G,H). In contrast, the total swimming distance and time in the target quadrant were significantly greater in the LMNXD-LD + SHR, LMNXD-MD + SHR, and MPH + SHR groups than in the SHR group (Figure 3G). The LMNXD-HD + SHR group covered a significantly longer distance in the target quadrant compared to the SHR group (*P* < 0.05, Figure 3H), although the increase in time spent there did not reach statistical significance. These findings demonstrate that LMNXD effectively enhances spatial memory in SHR/NCrl rats, with the medium dose yielding the most substantial therapeutic effect. Representative swimming paths for each group are presented in Figure 3I.

### 3.4 Effects of LMNXD on the expression levels of GFAP, DRD1, and BDNF in the brain tissue of SHR/NCrl rats

**FIGURE 4:**
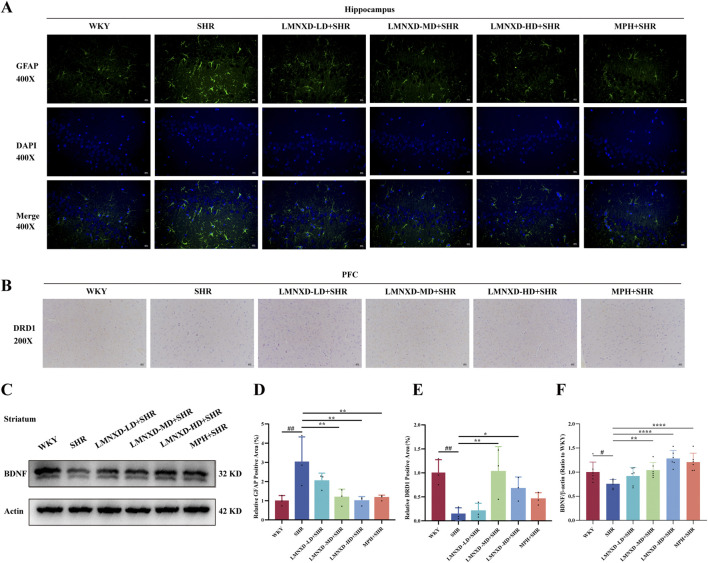
Effects of LMNXD on the relative expression of GFAP and DRD1 positive Area% in the hippocampus and PFC of rats and BDNF protein in the striatum. **(A)** Immunofluorescence staining of GFAP (Scale: 100um). **(B)** Immunohistochemical staining of DRD1 (Scale: 200um). **(C)** Western blotting analysis image of BDNF in the striatum. **(D)** Relative GFAP positive area%. **(E)** Relative DRD1 positive area%. **(F)** Relative expression of BDNF proteins. Data are expressed as mean ± standard deviation, n = 3 in immunohistochemistry and immunofluorescence, n = 6 in Western blot. Compared with the WKY group, ^#^ *p* < 0.05, ^##^ *p* < 0.01. Compared with the SHR group, ^*^ *p* < 0.05, ^**^ *p* < 0.01, ^****^ *p* < 0.0001.

GFAP serves as a classical marker of astrocyte activation. This activation promotes a pro-inflammatory state, which is significantly associated with the disease status and severity of ADHD. Immunofluorescence analysis evaluated the effect of LMNXD on the GFAP-positive area percentage in the rat hippocampus. The SHR group exhibited a significantly increased GFAP-positive area compared to the WKY group (*P* < 0.01) (Figures 4A,D). In contrast, the LMNXD-MD + SHR, LMNXD-HD + SHR, and MPH + SHR groups all showed a significant reduction relative to the SHR group (*P* < 0.01). Although the LMNXD-LD + SHR group also displayed a decrease, this difference was not statistically significant.

Distributed primarily in the PFC and striatum, DRD1 mediates excitatory G-protein signaling and regulates attention, impulse inhibition, and reward learning, making it a key node in ADHD-associated dopamine dysfunction. Immunohistochemistry assessed the impact of LMNXD on the DRD1-positive area percentage in the rat PFC. The DRD1-positive area was significantly lower in the SHR group than in the WKY group (*P* < 0.01) (Figures 4B,E). Compared to the SHR group, significant increases were observed in the LMNXD-MD + SHR (*P* < 0.01) and LMNXD-HD + SHR (*P* < 0.05) groups. The LMNXD-LD + SHR and MPH + SHR groups also showed increases, but these differences were not statistically significant.

As the most abundant neurotrophic factor in the central nervous system, BDNF regulates axonal branching, dendritic growth, and synaptic plasticity. Western blot analysis of striatal tissue revealed significantly lower BDNF protein expression in SHR rats compared to the WKY group (*P* < 0.05) (Figures 4C,F). BDNF protein expression was significantly increased in the LMNXD-MD + SHR, LMNXD-HD + SHR, and MPH + SHR groups relative to the SHR group (Figures 4C,F).

### 3.5 Network pharmacology untangles the targets and signaling pathways regulated by LMNXD

**FIGURE 5:**
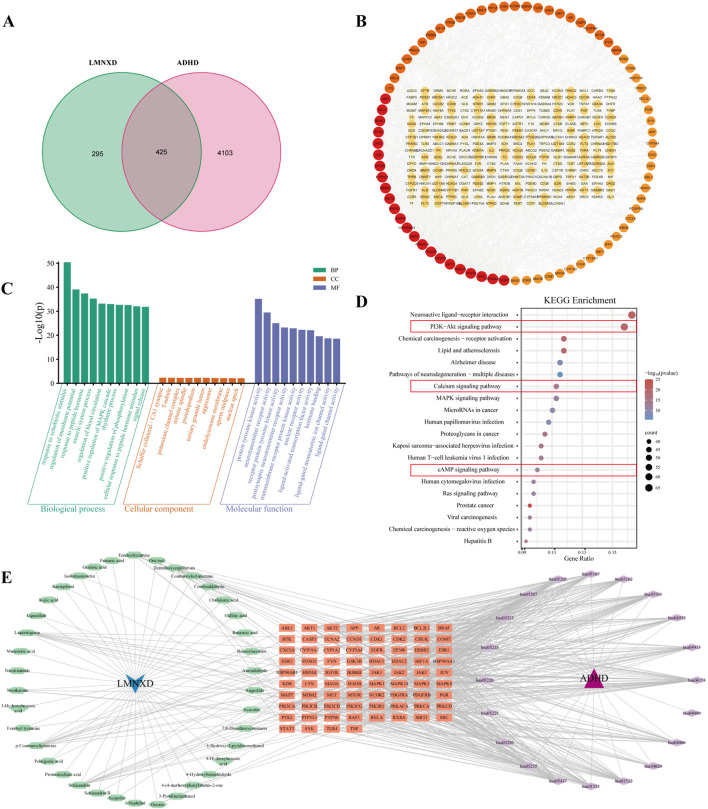
Network pharmacology untangles the targets and pathways regulated by LMNXD in ADHD. **(A)** Venn diagram of LMNXD active ingredient targets and ADHD disease targets. **(B)** PPI network of overlapping targets. **(C)** Triple plots of the top 10 enrichment results for each GO functional category: BP, cCC, and MF (*p* < 0.05). **(D)** Bubble plot of the top 20 enriched KEGG signaling pathways (*p* < 0.05). **(E)** LMNXD-Active Ingredients-Core Overlapping Targets-KEGG Signaling Pathway-ADHD network.

Seventy compounds identified through mass spectrometry were screened according to Lipinski’s Rule of Five, selecting those with at least two “Yes” outcomes and high gastrointestinal absorption, which yielded 45 candidate bioactive constituents (Supplementary Table 2). These constituents were submitted to the SwissTargetPrediction and STITCH databases for target prediction, yielding 722 unique targets after merging and deduplication. From the OMIM, GeneCards, DrugBank, and PharmGkb databases, 4528 ADHD-related targets were collected. A subsequent Venn diagram identified 425 potential targets implicated in LMNXD’s action against ADHD (Figure 5A). A PPI network constructed from these 425 overlapping targets contained 360 nodes and 1,287 edges (Figure 5B), where node size and color intensity reflected degree centrality. The outermost ring highlighted targets with a degree ≥10, among which PIK3R1, HSP90AA1, SRC, STAT3, PIK3CA, PIK3CB, AKT1, and PIK3CD emerged as potential core targets. GO and KEGG enrichment analyses performed *via* DAVID revealed that biological processes (BP) were mainly associated with xenobiotic stimulus response, membrane potential regulation, peptide hormone response, and muscle system processes. Key molecular functions (MF) encompassed protein tyrosine kinase activity, neurotransmitter receptor activity, transmembrane receptor protein tyrosine kinase activity, and postsynaptic neurotransmitter receptor activity. The principal cellular components (CC) included the Schaffer collateral–CA1 synapse, T-tubule, potassium channel complex, and mitotic spindle (Figure 5C). KEGG analysis highlighted significant enrichment in the PI3K–Akt, calcium, and cAMP signaling pathways (Figure 5D). An integrated “LMNXD–Constituents–Core Targets–Pathways–Disease” network, built using Cytoscape (Figure 5E), illustrated how individual constituents can modulate multiple targets and how multiple targets converge on common pathways. In summary, these network pharmacology results offer valuable mechanistic insight into LMNXD’s potential role in ADHD treatment.

### 3.6 Transcriptomic analysis of LMNXD treatment in SHR/NCrl rats

**FIGURE 6:**
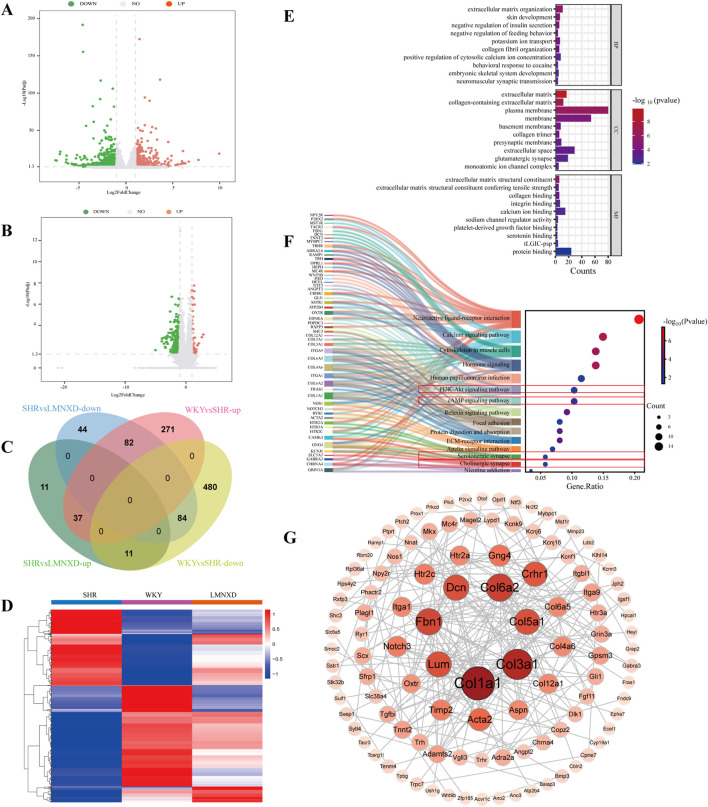
Transcriptome Sequencing Analysis of LMNXD in SHR/NCrl rats. **(A)** DEGs identified in hippocampal samples from the WKY and SHR groups. **(B)**Hippocampal DEGs between the SHR and LMNXD treatment groups. **(C)** Overlapof DEGs across the experimental groups. **(D)** Heatmap representation of the DEGs. **(E)** GO enrichment analysis for the overlapping DEGs. **(F)** KEGG pathway enrichment analysis of the overlapping DEGs. **(G)** PPI network constructed from the overlapping DEGs.

To investigate the biological mechanisms underlying LMNXD’s therapeutic effects, we conducted RNA sequencing on hippocampal tissues. The DEGs across the experimental groups are displayed in Figures 6A,B. Relative to the WKYgroup, the SHR group displayed 390 upregulated and 571 downregulated genes. Treatment with LMNXD resulted in 59 upregulated and 210 downregulated genes compared to the SHR group. We identified a total of 214 overlapping DEGs common to all three groups (Figures 6C,D). These 214 overlapping DEGs were subsequently subjected to GO, KEGG enrichment pathways, and PPI analyses. GO analysis indicated significant enrichment in processes and components including extracellular matrix organization, skin development, extracellular matrix, collagen-containing extracellular matrix, extracellular matrix structural constituent, extracellular matrix structural constituent conferring tensile strength. Figure 6E presents the ten most significantly enriched terms for BP, CC, and MF. KEGG pathway analysis further revealed significant enrichment in the cAMP, PI3K-Akt, Serotonergic synapse and Cholinergic synapses (Figure 5F). We performed PPI analysis using the STRING database and visualized the interaction network with Cytoscape software, yielding a network of 190 core nodes connected by 229 edges (Figure 6G). The DEGs were ranked according to the degree value of their corresponding nodes, which reflects their number of connections. This analysis identified Col1a1, Col3a1, Col6a2, Fbn1, and Dcn as the top five hub genes.

### 3.7 RT-qPCR validation of key targets in the PI3K-Akt and cAMP signaling pathways

**FIGURE 7:**
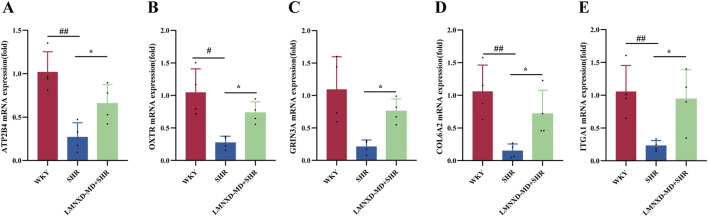
Effects of LMNXD on the relative mRNA expression of ATP2B4, OXTR, GRIN3A, ITGA1, and COL6A2 in the rat hippocampus. **(A)** Relative mRNA expression of ATP2B4. **(B)** Relative mRNA expression of OXTR. **(C)** Relative mRNA expression of GRIN3A. **(D)** Relative mRNA expression of COL6A2. **(E)** Relative mRNA expression of ITGA1 Data are presented as the mean ± standard deviation (n = 4 per group). ^#^ *p* < 0.05, ^##^ *p* < 0.01 compared with the WKY group; *p < 0.05 compared with the SHR group.

The PI3K-Akt and cAMP signaling pathways were significantly enriched in both network pharmacology and transcriptomics analyses, suggesting that they might be the key pathways for LMNXD in the treatment of ADHD. In addition, we screened out the genes enriched in these two pathways, with significant differences and related to the pathogenesis of ADHD from the transcriptome results, and conducted qPCR experiments for verification. The results indicated that the relative mRNA expression levels of ATP2B4, OXTR, GRIN3A, ITGA1, and COL6A2 were lower in the SHR group than in the WKY group. Conversely, the LMNXD-MD + SHR group exhibited significantly increased expression levels of these genes relative to the SHR group (Figures 7A–E).

## 4 Discussion

ADHD represents a common neurodevelopmental condition affecting children and adolescents. Pharmacotherapy, particularly first-line treatment with central nervous system stimulants, effectively manages core symptoms, though adverse effects such as sleep disturbances and cardiovascular risks during long-term use remain a concern (Shier et al., 2013; Storebø et al., 2023). Consequently, identifying novel therapeutic targets and developing comprehensive intervention strategies is essential. In this context, clinical use of natural product formulations to circumvent or reduce reliance on conventional psychotropic medications is becoming increasingly common.

This study preliminarily evaluated the efficacy of LMNXD in treating ADHD. Four-week-old male SHR/NCrl rats were selected as the ADHD model, corresponding developmentally to 9-year-old humans. The significantly higher incidence of ADHD in boys than in girls (Ayano et al., 2023). We therefore assessed the behavioral effects of LMNXD on 4-week-old male SHR using the OFT and MWM test. The OFT captured the characteristic hyperactivity and impulsivity of SHR/NCrl rats, providing rapid and reproducible behavioral indicators for drug screening (Moon et al., 2014; Zhang et al., 2022). The MWM test evaluated cognitive deficits in SHR/NCrl rats, focusing on spatial learning, memory, and cognitive flexibility within executive function (Xu et al., 2019). LMNXD treatment significantly reduced the total distance traveled and average velocity of SHR/NCrl rats in the OFT, with medium and high doses producing the most pronounced effects. During the MWM test spatial probe trial, the LMNXD-MD group exhibited a significant increase in platform crossings, target quadrant swimming distance, and time spent in the target quadrant.These results indicate that LMNXD significantly ameliorates hyperactive behavior and improves spatial memory in SHR/NCrl rats, with the medium-dose effect being the most obvious.

Elevated GFAP levels indicate astrocyte activation, a process closely linked to neuroinflammation, brain injury, and disrupted neurotransmitter homeostasis. These activated astrocytes release pro-inflammatory factors such as IL-1β and TNF-α, along with complement components like C3, which disturb the balance of key neurotransmitters including DA and NE and ultimately contribute to cognitive and behavioral deficits (Kwon and Koh, 2020; Borodinova et al., 2021). Multiple animal studies have confirmed a marked upregulation of GFAP expression in ADHD models. For example, rats with ADHD-like behaviors induced by chronic monosodium glutamate exposure showed increased GFAP expression alongside elevated pro-inflammatory cytokines IL-1β and TNF-α, as well as disturbances in neurotransmitters such as DA and NE (Abu-Elfotuh et al., 2022). DRD1 is regarded as an important candidate gene for ADHD, with numerous studies demonstrating that specific DRD1 single nucleotide polymorphisms and haplotypes significantly associate with ADHD susceptibility (Misener et al., 2004; Ribasés et al., 2012). By modulating dopaminergic signaling pathways, DRD1 influences PFC and striatal function, thereby contributing to the core symptoms of ADHD. For instance, a ketogenic diet can indirectly regulate DRD1 expression through gut microbiota alterations, increasing brain levels of serotonin and NE and subsequently improving ADHD-related behaviors (Liu et al., 2023). BDNF promotes neuronal survival, synaptic plasticity, and monoamine neurotransmitter transmission through its receptor tropomyosin receptor kinase B (TrkB) (Bazzari and Bazzari, 2022). Activation of the BDNF/TrkB pathway elevates DA levels in the synaptic cleft, which enhances behavioral performance. Previous work suggested that the TCM compound An Shen Ding Zhi Ling improves hyperactivity and cognitive deficits in an ADHD rat model by regulating both BDNF/TrkB and BDNF/P75/JNK1/NF-κB signaling pathways (Yaqun et al., 2022). In the present study, LMNXD-MD, LMNXD-HD, and MPH treatments markedly increased DRD1 and BDNF expression, while reducing GFAP expression in SHR rats.

Subsequently, we integrated network pharmacology with transcriptomic data to elucidate the molecular mechanisms underlying LMNXD’s therapeutic effects in ADHD. Target prediction was conducted for 70 chemical components of LMNXD identified through prior mass spectrometry, and these predicted targets were intersected with known disease-related targets, yielding 425 overlapping candidates. GO enrichment analysis suggested that LMNXD may act by modulating membrane potential, regulating neurotransmitter receptors, and influencing hippocampal synaptic function and ion channel activity. KEGG enrichment analysis further highlighted several key pathways, including the PI3K-Akt, calcium signaling, and cAMP signaling pathways.

Transcriptome sequencing of rat hippocampal tissues was then conducted to identify genes, biological processes, and pathways regulated by LMNXD in its treatment of ADHD. GO enrichment analysis indicated that LMNXD acts through multiple dimensions and pathways, such as extracellular matrix remodeling, synaptic transmission enhancement, ion channel regulation, and neurotransmitter balance restoration. These results align well with earlier network pharmacology findings. KEGG pathway analysis further demonstrated that LMNXD functions by modulating the cAMP signaling pathway, PI3K-Akt signaling pathway, serotonergic synapse, and cholinergic synapse. PPI analysis suggested Col1a1, Col3a1, Col6a2, Fbn1, and Dcn as potential core targets of LMNXD in treating the disease.

Studies have demonstrated that the cAMP signaling pathway plays a pivotal role in DA neurotransmission. This pathway activates adenylate cyclase (AC) and protein kinase A (PKA), which in turn regulate the expression of cAMP response element-binding protein (CREB) and BDNF(Brami-Cherrier et al., 2002; Li et al., 2024). It contributes to learning, memory formation, synaptic plasticity, and neuronal survival. For example, exercise significantly elevates hippocampal levels of BDNF, p-CREB, and TrkB proteins, resulting in improved spatial memory performance (Wu et al., 2020). Furthermore, activation of the cAMP/PKA/CREB-BDNF signaling pathway enhances mitochondrial dynamics and synaptic plasticity in mice while suppressing oxidative stress, ultimately improving learning and memory capabilities (Jiang et al., 2025).

The PI3K-Akt signaling pathway critically regulates neuronal development, synaptic plasticity, and neuroprotection, with its dysregulation linked to multiple neurodevelopmental disorders (Xiu et al., 2024). This pathway promotes neuronal survival and differentiation through the activation of specific downstream targets. Electrical stimulation, for instance, markedly increases the rate at which neural precursor cells differentiate into neurons by engaging PI3K-Akt signaling (Dong et al., 2019). Furthermore, the PI3K/Akt pathway supports the synthesis of synaptic proteins and the remodeling of synaptic architecture. BDNF activates the PI3K/Akt pathway to enhance synaptic plasticity, thereby improving learning and memory (Liu et al., 2025). The neuroprotective functions of this pathway are primarily expressed through anti-apoptotic, antioxidant, and anti-inflammatory mechanisms. In models of Alzheimer’s disease, PI3K-Akt pathway activation reduces neuronal apoptosis and slows disease progression (Bhatia et al., 2025).This pathway also activates nuclear factor erythroid 2-related factor 2 (Nrf2), which elevates antioxidant enzyme expression, and suppresses nuclear factor-kappa B (NF-κB) signaling to alleviate neuroinflammation (Hannan et al., 2020; Faysal et al., 2025).

Serotonergic and cholinergic synapses are both implicated in the pathophysiology of ADHD. Reduced levels of serotonin metabolites, such as 5-HIAA, have been observed in ADHD patients, indicating diminished global serotonin activity (Jackson et al., 2025). Furthermore, polymorphisms in the serotonin synthesis enzyme gene TPH2 and the serotonin transporter gene SERT show significant associations with impulsive symptoms in the disorder (Oades, 2008). Serotonin modulates other neurotransmitter systems through its receptors, for example, by inhibiting DA release or enhancing NE effects, which in turn affects attention control and behavioral inhibition (Burke et al., 2014). Cholinergic synapses regulate cognitive functions through nicotinic and muscarinic acetylcholine receptors (nAChRs and mAChRs). Decreased acetylcholine (ACh) levels in the PFC can impair working memory and attention (Wallace and Bertrand, 2013). Stimulating α4β2 nAChRs increases dopaminergic neuron firing, which enhances prefrontal dopamine transmission and supports executive function (Wilens and Decker, 2007). Atomoxetine indirectly elevates ACh release in the PFC by activating α1-adrenergic receptors, resulting in improved cognitive performance (Tzavara et al., 2006). Gastrodin alleviates ADHD symptoms by modulating cholinergic synapses, such as *via* CHRNA3 receptor activation, and dopaminergic synapses, including through DRD2/DRD4 receptors, producing synergistic therapeutic outcomes (Song et al., 2022).

Given the significant enrichment of both network pharmacology and transcriptomic analyses in the cAMP and PI3K-Akt signaling pathways, which suggests their central role in the mechanism of LMNXD for treating ADHD, we validated the genes enriched in these pathways using qPCR. The results showed that, compared with the WKY group, the expression levels of ATP2B4, OXTR, GRIN3A, COL6A2, and ITGA1 were downregulated in the SHR group, while LMNXD treatment reversed these changes. This trend aligns with the findings from the transcriptomic analysis.

ATP2B4 mediates the transmembrane transport of calcium ions by actively extruding intracellular Ca^2+^, which prevents excessive accumulation following synaptic activity and protects neurons from calcium overload-induced toxicity. This protein can form a complex with nNOS to modulate local cGMP levels, thereby influencing the cAMP/PKA signaling pathway (Mohamed et al., 2011). Research indicates that ATP2B4 is essential for neural stem cells to resist oxidative stress; its upregulation significantly enhances oxidative stress tolerance, improves cell viability, and suppresses apoptosis (Nie et al., 2024). As calcium signaling critically regulates neurotransmitter release, ATP2B4 rapidly clears intracellular calcium to terminate neurotransmitter release and prevent excessive synaptic activation (Ghosh et al., 2011).

As a member of the G protein-coupled receptor family, OXTR activates or inhibits AC through Gαs or Gαi/o signaling pathways, respectively, thereby modulating the cAMP/PKA/CREB signaling axis (Walker et al., 2022). In a methamphetamine (METH)-induced neurotoxicity model, oxytocin (OT) pretreatment to activate OXTR reversed the METH-induced reduction in p-CREB expression, upregulated the anti-apoptotic protein Bcl-2, and downregulated the pro-apoptotic protein Bax, ultimately suppressing hippocampal neuronal apoptosis (Li et al., 2021). These results indicate that OXTR-mediated signaling protects against METH-induced neuronal injury.

GRIN3A, a key regulatory subunit of the NMDA-type glutamate receptor (NMDAR), displays a marked age-dependent expression pattern. Its expression peaks during early development and progressively declines as neurons mature. This downregulation of GRIN3A represents a critical step in synaptic functional maturation. Premature or delayed reduction can impair synaptic plasticity and disrupt higher cognitive functions, including learning and memory (Murillo et al., 2021; Choi et al., 2022). Clinical studies have further demonstrated significantly reduced GRIN3A expression in the hippocampi of Alzheimer’s disease patients, which compromises postsynaptic function (Lee et al., 2024). GRIN3A also contributes to presynaptic regulation. Research indicates that interferon alpha enhances NMDA/glutamate-triggered neurotransmitter release from synaptosomes by inducing GRIN3A expression (Obolenskaya et al., 2021). Together, these findings underscore the need for deeper investigation into the regulatory role of GRIN3A in neurodevelopment and neurodegenerative diseases.

COL6A2, a key component of the extracellular matrix (ECM), exerts significant anti-apoptotic effects in the nervous system. Under stress conditions, hippocampal neurons upregulate COL6A2 expression. This upregulation activates the Akt/PI3K pathway, which enhances anti-apoptotic capacity, reduces oxidative stress damage, and maintains neuronal synaptic stability (Cheng et al., 2011). The absence of COL6A2 disrupts dopaminergic neural circuit development and function, leading to sensory-motor gating deficits, impaired working memory, and attention deficits (Gregorio et al., 2022). ITGA1, another critical ECM receptor, modulates neural synaptic plasticity and dopaminergic circuit function by regulating cell adhesion, migration, and signal transduction (Liu et al., 2017). Hypobilirubinemia represents a pathological state linked to multiple neurodevelopmental and neurodegenerative disorders. In a hypobilirubinemia model, elevated ITGA1 expression contributed to neuroinflammation and neuronal structural damage *via* activation of the PI3K-Akt signaling pathway (Chen et al., 2025).

### 4.1 Limitations and prospects

This study has several limitations. First, the limited sample size and considerable dispersion in some data may have reduced statistical power, potentially preventing certain intergroup differences from reaching significance. Second, the validation of DEGs was relatively narrow; subsequent work should confirm these findings at the protein level using methods such as Western blot analysis. Third, although this study provides preliminary evidence for the potential involvement of the cAMP and PI3K-Akt signaling pathways, the complete upstream and downstream molecular mechanisms still require systematic investigation. Finally, while this research examined the overall therapeutic effect of the compound formulation, the specific active constituents responsible for its efficacy remain to be identified.

## 5 Conclusion

In summary, our findings demonstrate that LMNXD ameliorates hyperactivity, impulsivity, spatial memory, and learning ability in SHR rats, while effectively reducing hippocampal GFAP expression and increasing DRD1 expression in the PFC alongside BDNF expression in the striatum. Through the integration of network pharmacology and transcriptomics, we preliminarily identified potential mechanisms for LMNXD in treating ADHD. These results suggest that LMNXD may exert its therapeutic effects by modulating proteins associated with the cAMP and PI3K-Akt signaling pathways.

## Data Availability

The datasets presented in this study can be found in online repositories. The names of the repository/repositories and accession number(s) can be found below: https://ngdc.cncb.ac.cn/gsa/, CRA033160.
