# Supplementary material for: Integrating network pharmacology and transcriptomics to reveal the therapeutic effect of Long Mu Ning Xin Decoction on attention-deficit/hyperactivity disorder by regulating cAMP and PI3K/AKT pathways
Source: Front Pharmacol. 2026 Jan 30;17:1744709. doi: 10.3389/fphar.2026.1744709 (PMC12901447; doi:10.3389/fphar.2026.1744709)
Supplement: Supplementary file 1 [file Table1.docx]

**Supplementary Material**

**Supplementary Table 1 The chemical components in LMNXD were identified based on UHPLC-OE-MS technology**

| NO. | Compound Name | Compound Class | Formula | m/z | Rt/min | Mass Error （PPM） | mz2 Adduct | PubChem ID | Confidence Level |
| --- | --- | --- | --- | --- | --- | --- | --- | --- | --- |
| 1 | Galactose | Saccharides | C6H12O6 | 203.0527 | 45.1 | 0.2 | [M+Na]+ | 86278404 | level1 |
| 2 | Tagatose | Saccharides | C6H12O6 | 203.0527 | 45.1 | 0.2 | [M+Na]+ | 92092 | level1 |
| 3 | Schisandrin B | Lignans | C23H28O6 | 401.1961 | 483.3 | 0.6 | [M+H]+ | 108130 | level1 |
| 4 | Demethoxycapillarisin | Coumarins | C15H10O6 | 287.0551 | 372.8 | 0 | [M+H]+ | 5316511 | level1 |
| 5 | Liquiritigenin | Flavonoids | C15H12O4 | 257.0809 | 341.2 | 0.3 | [M+H]+ | 114829 | level1 |
| 6 | N-p-trans-Coumaroyltyramine | Phenylpropanoids (C6-C3) | C17H17NO3 | 284.1282 | 329 | 0.2 | [M+H]+ | 5372945 | level1 |
| 7 | Schisandrin | Lignans | C24H32O7 | 433.222 | 385.8 | 0.3 | [M+H]+ | 23915 | level1 |
| 8 | N-trans-Feruloyltyramine | Phenylpropanoids (C6-C3) | C18H19NO4 | 314.1389 | 326.4 | 0.5 | [M+H]+ | 5280537 | level1 |
| 9 | Ononin | Isoflavonoids | C22H22O9 | 431.134 | 321.2 | 0.7 | [M+H]+ | 442813 | level1 |
| 10 | 3,5-dimethoxy-4-[(2S,3R,4S,5S,6R)-3,4,5-trihydroxy-6-(hydroxymethyl)tetrahydropyran-2-yl]oxy-benzoic acid | Phenolic acids (C6-C1) | C15H20O10 | 359.0981 | 218 | 0.6 | [M-H]- | 10383888 | level1 |
| 11 | 6-(hydroxymethyl)pyridin-3-ol | Nicotinic acid alkaloids | C6H7NO2 | 108.0445 | 65.2 | 0.7 | [M-H2O+H]+ | 419490 | level1 |
| 12 | 3-Pyridinemethanol | Nicotinic acid alkaloids | C6H7NO | 110.0601 | 51.6 | 0.2 | [M+H]+ | 7510 | level1 |
| 13 | Trigonelline | Nicotinic acid alkaloids | C7H7NO2 | 138.055 | 47.4 | 0.4 | [M+H]+ | 5570 | level1 |
| 14 | Angeolide | Cyclic polyketides | C24H28O4 | 381.2063 | 443.5 | 0.5 | [M+H]+ | 54607915 | level1 |
| 15 | Kaempferol | Flavonoids | C15H10O6 | 285.04 | 374 | 1.6 | [M-H]- | 5280863 | level1 |
| 16 | Asaraldehyde |  | C10H12O4 | 197.0809 | 316.7 | 0 | [M+H]+ | 20525 | level1 |
| 17 | 7,8-dimethoxychromen-2-one | Coumarins | C11H10O4 | 207.0653 | 306.9 | 0.5 | [M+H]+ | 142768 | level1 |
| 18 | Polygalaxanthone III | Xanthones | C25H28O15 | 569.1504 | 280.7 | 0.4 | [M+H]+ | 11169063 | level1 |
| 19 | 4-Hydroxybenzaldehyde | Phenolic acids (C6-C1) | C7H6O2 | 121.0293 | 274 | 1.4 | [M-H]- | 126 | level1 |
| 20 | (2R,3R,4S,5R,6R)-2-(hydroxymethyl)-6-[[(2R,3S,4S,5R,6S)-3,4,5-trihydroxy-6-[[(1S,2S,4S,5S,6R,10S)-5-hydroxy-2-(hydroxymethyl)-3,9-dioxatricyclo[4.4.0.02,4]dec-7-en-10-yl]oxy]tetrahydropyran-2-yl]methoxy]tetrahydropyran-3,4,5-triol | Monoterpenoids | C21H32O15 | 523.1661 | 196.3 | 1.3 | [M-H]- | 101787412 | level1 |
| 21 | Linoleic acid | Fatty Acids and Conjugates | C18H32O2 | 279.2325 | 553.8 | 1.5 | [M-H]- | 5280450 | level1 |
| 22 | alpha-Linolenic acid | Fatty Acids and Conjugates | C18H30O2 | 277.2168 | 536.5 | 1.7 | [M-H]- | 5280934 | level1 |
| 23 | gamma-Linolenic acid | Fatty Acids and Conjugates | C18H30O2 | 277.2168 | 536.5 | 1.7 | [M-H]- | 5280933 | level1 |
| 24 | 7-hydroxy-2-(4-methoxyphenyl)chromen-4-one | Flavonoids | C16H12O4 | 269.0809 | 384.5 | 0.3 | [M+H]+ | 5320693 | level1 |
| 25 | Tenuifoliside A |  | C31H38O17 | 665.208 | 311.1 | 0.6 | [M-H2O+H]+ | 46933844 | level1 |
| 26 | Coniferaldehyde | Phenylpropanoids (C6-C3) | C10H10O3 | 179.0704 | 305.5 | 0.4 | [M+H]+ | 5280536 | level1 |
| 27 | Butanoic acid | Fatty Acids and Conjugates | C4H8O2 | 87.045 | 241.1 | 1.3 | [M-H]- | 264 | level1 |
| 28 | Hesperetin 7-O-neohesperidoside | Flavonoids | C28H34O15 | 609.1814 | 298.3 | 1.8 | [M-H]- | 442439 | level1 |
| 29 | 7-hydroxy-2-(4-hydroxyphenyl)chroman-4-one | Flavonoids | C15H12O4 | 257.0809 | 341.2 | 0.3 | [M+H]+ | 1889 | level1 |
| 30 | Nootkatone | Sesquiterpenoids | C15H22O | 219.1743 | 461.1 | 0.2 | [M+H]+ | 1268142 | level1 |
| 31 | Pelargonic acid | Fatty Acids and Conjugates | C9H18O2 | 157.1231 | 457.2 | 1.9 | [M-H]- | 8158 | level1 |
| 32 | Ligustilide | Cyclic polyketides | C12H14O2 | 191.1067 | 428.3 | 0.3 | [M+H]+ | 5319022 | level1 |
| 33 | 4-(4-methoxyphenyl)butan-2-one |  | C11H14O2 | 177.0917 | 426.7 | 2.2 | [M-H]- | 61007 | level1 |
| 34 | Magnesium Lithospermate B | Lignans | C36H30O16 | 717.1447 | 410 | 1.9 | [M-H]- | 5316299 | level1 |
| 35 | 2-(4-hydroxyphenyl)ethyl (E)-3-(4-hydroxy-3-methoxy-phenyl)prop-2-enoate | Phenylpropanoids (C6-C3) | C18H18O5 | 313.1077 | 379.4 | 1.2 | [M-H]- | 637308 | level1 |
| 36 | 7-hydroxy-2-[4-[(2S,3R,4S,5S,6R)-3,4,5-trihydroxy-6-(hydroxymethyl)tetrahydropyran-2-yl]oxyphenyl]chroman-4-one | Flavonoids | C21H22O9 | 417.1183 | 289.2 | 1.8 | [M-H]- | 11972398 | level1 |
| 37 | 2-[(2S,3R,4S,5S,6R)-3-[(2S,3R,4R)-3,4-dihydroxy-4-(hydroxymethyl)tetrahydrofuran-2-yl]oxy-4,5-dihydroxy-6-(hydroxymethyl)tetrahydropyran-2-yl]-1,3,7-trihydroxy-xanthen-9-one | Xanthones | C24H26O14 | 539.1399 | 281.5 | 0.6 | [M+H]+ | 21581293 | level1 |
| 38 | Vicenin-1 | Flavonoids | C26H28O14 | 563.1391 | 281.5 | 2.6 | [M-H]- | 13644663 | level1 |
| 39 | Syringaldehyde | Phenolic acids (C6-C1) | C9H10O4 | 183.0653 | 278.3 | 0.4 | [M+H]+ | 8655 | level1 |
| 40 | 5-methyl-4-[(2S,3R,4S,5S,6R)-3,4,5-trihydroxy-6-(hydroxymethyl)tetrahydropyran-2-yl]oxy-chromen-2-one | Coumarins | C16H18O8 | 339.1078 | 278.1 | 1.1 | [M+H]+ | 196468 | level1 |
| 41 | [(2R,3R,4R,5R,6R)-6-[2-(3,4-dihydroxyphenyl)ethoxy]-5-hydroxy-2-[[(2R,3R,4S,5S,6R)-3,4,5-trihydroxy-6-(hydroxymethyl)tetrahydropyran-2-yl]oxymethyl]-4-[(2S,3R,4R,5R,6S)-3,4,5-trihydroxy-6-methyl-tetrahydropyran-2-yl]oxy-tetrahydropyran-3-yl] (E)-3-(4-hydroxy-3-methoxy-phenyl)prop-2-enoate | Phenylpropanoids (C6-C3) | C36H48O20 | 799.2662 | 262.9 | 0.5 | [M-H]- | 6325450 | level1 |
| 42 | 4-Hydroxybenzoic acid | Phenolic acids (C6-C1) | C7H6O3 | 137.0242 | 255.7 | 1.2 | [M-H]- | 135 | level1 |
| 43 | Sibiricose A5 | Phenylpropanoids (C6-C3) | C22H30O14 | 517.1554 | 243.9 | 1.6 | [M-H]- | 6326020 | level1 |
| 44 | [(2R,3S,4S,5R,6R)-6-[(2S,3S,4S,5R)-3,4-dihydroxy-2,5-bis(hydroxymethyl)tetrahydrofuran-2-yl]oxy-3,4,5-trihydroxy-tetrahydropyran-2-yl]methyl 4-hydroxybenzoate | Phenolic acids (C6-C1) | C19H26O13 | 461.1293 | 232.6 | 1.5 | [M-H]- | 10813903 | level1 |
| 45 | (2S,3R,4S,5S,6R)-2-[[(1S,4aS,5R,7aR)-4a,5-dihydroxy-7-(hydroxymethyl)-5,7a-dihydro-1H-cyclopenta[c]pyran-1-yl]oxy]-6-(hydroxymethyl)tetrahydropyran-3,4,5-triol | Monoterpenoids | C15H22O10 | 361.1136 | 141.6 | 1.1 | [M-H]- | 11968396 | level1 |
| 46 | Chelidonic acid |  | C7H4O6 | 182.9932 | 103.2 | 1.4 | [M-H]- | 7431 | level1 |
| 47 | Kojic acid | Cyclic polyketides | C6H6O4 | 143.0341 | 98.6 | 1.1 | [M+H]+ | 3840 | level1 |
| 48 | Fumaric acid | Fatty Acids and Conjugates | C4H4O4 | 115.0035 | 79.3 | 1.5 | [M-H]- | 444972 | level1 |
| 49 | 3,4-Dihydroxybenzoic acid | Phenolic acids (C6-C1) | C7H6O4 | 153.0191 | 227.7 | 1.6 | [M-H]- | 72 | level1 |
| 50 | (1S,2S,6Z,10S,11S,16Z)-6,16-di(butylidene)-5,15-dioxapentacyclo[9.5.2.01,13.02,10.03,7]octadeca-3(7),12-diene-4,14-dione | Cyclic polyketides | C24H28O4 | 381.2062 | 468.8 | 0.5 | [M+H]+ | 70698035 | level1 |
| 51 | 3,4,5-Trimethoxybenzaldehyde | Lignans | C10H12O4 | 197.0809 | 316.7 | 0 | [M+H]+ | 6858 | level1 |
| 52 | (1R,7R,10R)-4,10,11,11-tetramethyltricyclo[5.3.1.01,5]undec-4-en-3-one | Sesquiterpenoids | C15H22O | 219.1743 | 461.1 | 0.2 | [M+H]+ | 12308615 | level1 |
| 53 | (2R)-2-[(E)-3-[3-[(1R)-1-carboxy-2-(3,4-dihydroxyphenyl)ethoxy]carbonyl-2-(3,4-dihydroxyphenyl)-7-hydroxy-2,3-dihydrobenzofuran-4-yl]prop-2-enoyl]oxy-3-(3,4-dihydroxyphenyl)propanoic acid | Lignans | C36H30O16 | 717.1447 | 410 | 1.9 | [M-H]- | 13991589 | level1 |
| 54 | Gentisic acid | Phenolic acids (C6-C1) | C7H6O4 | 153.0191 | 227.7 | 1.6 | [M-H]- | 3469 | level1 |
| 55 | (E)-5-(2,3-dimethyl-3-tricyclo[2.2.1.02,6]heptanyl)-2-methyl-pent-2-enoic acid | Sesquiterpenoids | C15H22O2 | 233.1543 | 502.5 | 1.5 | [M-H]- | 14059029 | level1 |
| 56 | 1-phenylbutane-1,3-dione |  | C10H10O2 | 163.0754 | 462 | 0.1 | [M+H]+ | 7166 | level1 |
| 57 | 1-Naphthol | Naphthalenes | C10H8O | 143.0499 | 384.9 | 2 | [M-H]- | 7005 | level1 |
| 58 | Rosmarinic acid | Phenylpropanoids (C6-C3) | C18H16O8 | 359.0765 | 311.4 | 2.1 | [M-H]- | 5281792 | level1 |
| 59 | N-p-Coumaroyloctopamine | Phenylpropanoids (C6-C3) | C17H17NO4 | 282.1127 | 293.5 | 0.8 | [M-H2O+H]+ | 23874492 | level1 |
| 60 | DL-3-Phenyllactic acid |  | C9H10O3 | 165.0554 | 287.9 | 1.8 | [M-H]- | 3848 | level1 |
| 61 | (E)-3',6-Disinapoylsucrose | Phenylpropanoids (C6-C3) | C34H42O19 | 755.2387 | 286.6 | 0.8 | [M+H]+ | 11968389 | level1 |
| 62 | (2E,4E)-5-[8-hydroxy-1,5-dimethyl-3-[3,4,5-trihydroxy-6-(hydroxymethyl)tetrahydropyran-2-yl]oxy-6-oxabicyclo[3.2.1]octan-8-yl]-3-methyl-penta-2,4-dienoic acid | Apocarotenoids | C21H32O10 | 443.1919 | 234.4 | 0.8 | [M-H]- | 129008896 | level1 |
| 63 | Nicotinamide | Nicotinic acid alkaloids | C6H6N2O | 123.0555 | 100 | 1.2 | [M+H]+ | 936 | level1 |
| 64 | Oricinol |  | C7H8O2 | 123.045 | 274.3 | 1.5 | [M-H]- | 10436 | level1 |
| 65 | Caffeic acid | Phenylpropanoids (C6-C3) | C9H8O4 | 179.0348 | 268.4 | 1.1 | [M-H]- | 689043 | level1 |
| 66 | Hesperidin | Flavonoids | C28H34O15 | 609.1814 | 298.3 | 1.8 | [M-H]- | 10621 | level1 |
| 67 | Isoformononetin | Isoflavonoids | C16H12O4 | 269.0809 | 384.5 | 0.3 | [M+H]+ | 3764 | level1 |
| 68 | 2-(2-phenylethoxy)-6-[(3,4,5-trihydroxytetrahydropyran-2-yl)oxymethyl]tetrahydropyran-3,4,5-triol | Phenylethanoids (C6-C2) | C19H28O10 | 415.1602 | 271.1 | 1.7 | [M-H]- | 14704521 | level1 |
| 69 | 3-Hydroxybenzoic acid | Phenolic acids (C6-C1) | C7H6O3 | 137.0242 | 270.9 | 1.7 | [M-H]- | 7420 | level1 |
| 70 | 2,3-Dihydroxybenzoic acid | Phenolic acids (C6-C1) | C7H6O4 | 153.0191 | 227.7 | 1.6 | [M-H]- | 19 | level1 |

**Supplementary Table 2 45 potential bioactive components**

| **PubChem ID** | **Compound Name** | **GI absorption** | **Lipinski** | **Ghose** | **Veber** | **Egan** | **Muegge** |
| --- | --- | --- | --- | --- | --- | --- | --- |
| 108130 | Schisandrin B | High | Yes | Yes | Yes | Yes | No |
| 5316511 | Demethoxycapillarisin | High | Yes | Yes | Yes | Yes | Yes |
| 114829 | Liquiritigenin | High | Yes | Yes | Yes | Yes | Yes |
| 5372945 | N-p-trans-Coumaroyltyramine | High | Yes | Yes | Yes | Yes | Yes |
| 23915 | Schisandrin | High | Yes | Yes | Yes | Yes | Yes |
| 5280537 | N-trans-Feruloyltyramine | High | Yes | Yes | Yes | Yes | Yes |
| 442813 | Ononin | High | Yes | Yes | Yes | No | Yes |
| 419490 | 6-(hydroxymethyl)pyridin-3-ol | High | Yes | No | Yes | Yes | No |
| 7510 | 3-Pyridinemethanol | High | Yes | No | Yes | Yes | No |
| 5570 | Trigonelline | High | Yes | No | Yes | Yes | No |
| 54607915 | Angeolide | High | Yes | Yes | Yes | Yes | Yes |
| 5280863 | Kaempferol | High | Yes | Yes | Yes | Yes | Yes |
| 20525 | Asaraldehyde | High | Yes | Yes | Yes | Yes | No |
| 142768 | 7,8-dimethoxychromen-2-one | High | Yes | Yes | Yes | Yes | Yes |
| 126 | 4-Hydroxybenzaldehyde | High | Yes | No | Yes | Yes | No |
| 5320693 | 7-hydroxy-2-(4-methoxyphenyl)chromen-4-one | High | Yes | Yes | Yes | Yes | Yes |
| 5280536 | Coniferaldehyde | High | Yes | Yes | Yes | Yes | No |
| 264 | Butanoic acid | High | Yes | No | Yes | Yes | No |
| 1889 | 7-hydroxy-2-(4-hydroxyphenyl)chroman-4-one | High | Yes | Yes | Yes | Yes | Yes |
| 1268142 | Nootkatone | High | Yes | Yes | Yes | Yes | No |
| 8158 | Pelargonic acid | High | Yes | No | Yes | Yes | No |
| 5319022 | Ligustilide | High | Yes | Yes | Yes | Yes | No |
| 61007 | 4-(4-methoxyphenyl)butan-2-one | High | Yes | Yes | Yes | Yes | No |
| 637308 | 2-(4-hydroxyphenyl)ethyl (E)-3-(4-hydroxy-3-methoxy-phenyl)prop-2-enoate | High | Yes | Yes | Yes | Yes | Yes |
| 8655 | Syringaldehyde | High | Yes | Yes | Yes | Yes | No |
| 196468 | 5-methyl-4-[(2S,3R,4S,5S,6R)-3,4,5-trihydroxy-6-(hydroxymethyl)tetrahydropyran-2-yl]oxy-chromen-2-one | High | Yes | No | Yes | Yes | Yes |
| 135 | 4-Hydroxybenzoic acid | High | Yes | No | Yes | Yes | No |
| 7431 | Chelidonic acid | High | Yes | No | Yes | Yes | No |
| 3840 | Kojic acid | High | Yes | No | Yes | Yes | No |
| 444972 | Fumaric acid | High | Yes | No | Yes | Yes | No |
| 72 | 3,4-Dihydroxybenzoic acid | High | Yes | No | Yes | Yes | No |
| 6858 | 3,4,5-Trimethoxybenzaldehyde | High | Yes | Yes | Yes | Yes | No |
| 12308615 | (1R,7R,10R)-4,10,11,11-tetramethyltricyclo[5.3.1.01,5]undec-4-en-3-one | High | Yes | Yes | Yes | Yes | No |
| 3469 | Gentisic acid | High | Yes | No | Yes | Yes | No |
| 14059029 | (E)-5-(2,3-dimethyl-3-tricyclo[2.2.1.02,6]heptanyl)-2-methyl-pent-2-enoic acid | High | Yes | Yes | Yes | Yes | Yes |
| 7166 | 1-phenylbutane-1,3-dione | High | Yes | Yes | Yes | Yes | No |
| 7005 | 1-Naphthol | High | Yes | No | Yes | Yes | No |
| 23874492 | N-p-Coumaroyloctopamine | High | Yes | Yes | Yes | Yes | Yes |
| 3848 | DL-3-Phenyllactic acid | High | Yes | Yes | Yes | Yes | No |
| 936 | Nicotinamide | High | Yes | No | Yes | Yes | No |
| 10436 | Oricinol | High | Yes | No | Yes | Yes | No |
| 689043 | Caffeic acid | High | Yes | Yes | Yes | Yes | No |
| 3764 | Isoformononetin | High | Yes | Yes | Yes | Yes | Yes |
| 7420 | 3-Hydroxybenzoic acid | High | Yes | No | Yes | Yes | No |
| 19 | 2,3-Dihydroxybenzoic acid | High | Yes | No | Yes | Yes | No |
